# VPS72, a member of VPS protein family, can be used as a new prognostic marker for hepatocellular carcinoma

**DOI:** 10.1002/iid3.856

**Published:** 2023-05-22

**Authors:** Jian Huang, Jin Gan, Jian Wang, Min Zheng, Han Xiao

**Affiliations:** ^1^ Department of General surgery Jiujiang First People's Hospital Jiujiang China; ^2^ Department of Hepato‐Biliary‐Pancreatic Surgery Pingxiang People's Hospital Pingxiang China; ^3^ Department of rehabilitation Lushan People's Hospital Jiujiang China; ^4^ Department of Hepato‐Biliary‐Pancreatic Surgery Jiujiang First People's Hospital Jiujiang China

**Keywords:** bioinformatics, hepatocellular carcinoma, vacuolar protein sorting family, VPS72

## Abstract

**Background:**

Vacuolar protein sorting (VPS) plays a crucial role in intracellular molecular transport between organelles. However, studies have indicated a correlation between VPSs and tumorigenesis and the development of several cancers. Nevertheless, the association between VPSs and hepatocellular carcinoma (HCC) remains unclear.

**Methods:**

By analyzing databases such as The Cancer Genome Atlas (TCGA) and The International Cancer Genome Consortium (ICGC), we investigated the differences in VPSs expression between normal tissue and HCC transcriptomes. Furthermore, we examined the relationship between VPSs expression and overall survival (OS) in patients with HCC. Univariate and multivariate Cox analyses were employed to assess the prognostic value of VPS72 as an independent factor, and the correlation between VPS72 and the tumor immune microenvironment was also analyzed.

**Results:**

We observed significant overexpression of 28 VPSs in HCC tissues compared to normal tissues. The mRNA expression of VPSs displayed a negative correlation with OS, while exhibiting a positive correlation with tumor grade and stage. Additionally, both univariate and multivariate Cox analyses identified VPS72 as a potential independent risk factor for HCC prognosis. Overexpression of VPS72 demonstrated a positive correlation with various clinicopathological factors associated with poor prognosis, as well as the infiltration levels of immune cells.

**Conclusion:**

Therefore, our research shows that VPSs participate in HCC occurrence and development, especially VPS72, which may act as a potential target for HCC treatment and prognosis biomarker.

## Graphical abstract

Our research shows that vacuolar protein sortings (VPSs) play an essential role in the occurrence and development of hepatocellular carcinoma (HCC), especially VPS72 can be used as a potential target for treatment and prognosis biomarker of HCC.

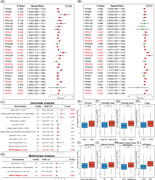

## 1 INTRODUCTION

Hepatocellular carcinoma (HCC) ranked the sixth most prevalent malignancy and the third major cause of cancer‐related mortalities globally in 2020, with about 906,000 new cases and 830,000 deaths.^1^ Regarding HCC treatment, surgical resection can be an option for only 5%–15% of patients at early stages, while the advanced treatment depends on transcatheter arterial chemoembolization (TACE) or oral sorafenib.^2^ In recent years, immunotherapy and molecularly targeted therapy research have advanced rapidly, with the combined anti‐PD‐L1 (programmed cell death‐Ligand 1) antibody atezolizumab and VEGF（vascular endothelial growth factor）‐neutralizing antibody bevacizumab becoming the standard first‐line therapy for HCC,^3^ but the effect of immunotherapy on nonalcoholic steatohepatitis (NASH) and HCC is completely unclear, so the application of immunotherapy in HCC has not achieved very satisfactory results.^4^ HCC has various molecular pathogenesis; HCC has many potential operable mutations, but these mutations have not been translated into clinical practice.^5^ Therefore, through the discovery of new biomarkers of diagnosis and treatment, we can better understand the complex relationship between HCC occurrence and development and the immune microenvironment, as well as improve patient prognosis and individualized treatment.

In eukaryotic cells, membrane‐wrapped vesicles are involved in the transport of intracellular molecules along organelles, including the endoplasmic reticulum (ER), Golgi apparatus, endosome, secretory vesicles, and lysosomes.^6^, ^7^ Functionally, vacuolar protein sorting (VPS) is crucial for decarbonizing in the endocytosis, biosynthesis and secretion, exocytosis, and recovery of intracellular molecules.^8^, ^9^, ^10^ Among them, VPSs play a significant role in mammalian vesicle transport.^11^ There are 30 human VPS genes in the GeneCards database (www.genecards.org). There is only a small amount of research on the association between VPSs genes and their products and human diseases. Many physiological processes in the human body are affected by abnormal VPS expression, such as ubiquitin disruption in the signal transduction pathway following VPS11/VPS18 overexpression.^12^ In the absence of VPS13, not only the cortical ER marker Rtn1 accumulation in late endosomes is observed, but also a sharp reduction in the number of ER packaged into autophagosomes.^13^ The abnormal expression of VPS is related to some human neurodegenerative diseases. For example, VPS41 loss is linked to human progressive neurodevelopmental disorders.^14^ At the same time, some VPS family members' abnormal expression and prognostic values have also been discovered in tumor‐related studies. In gastric cancer, VPS52 is a tumor suppressor gene, and its absence is correlated to a poor prognosis. VPS52 overexpression inhibits tumor proliferation both in vitro and in vivo.^15^ Wang et al. found that VPS33b is a crucial tumor suppressor in hepatocellular carcinogenesis, and its downregulation is a key step in inflammation‐driven HCC^16^; however, the role of the human VPS gene and its products in malignancies is largely unknown. Recently, with the ongoing advancement of microarray technology and bioinformatics, many sequencing data have been produced. The analysis of thousands of gene expression or copy number variations available in the online database was used for examining the correlation between different VPSs expressions and clinical parameters, including the overall survival (OS) rate of HCC patients; moreover, VPSs role in HCC occurrence and development was investigated.

## 2 RESULTS

### 2.1 Transcriptional levels of VPSs family between HCC and normal tissues

**Figure 1:**
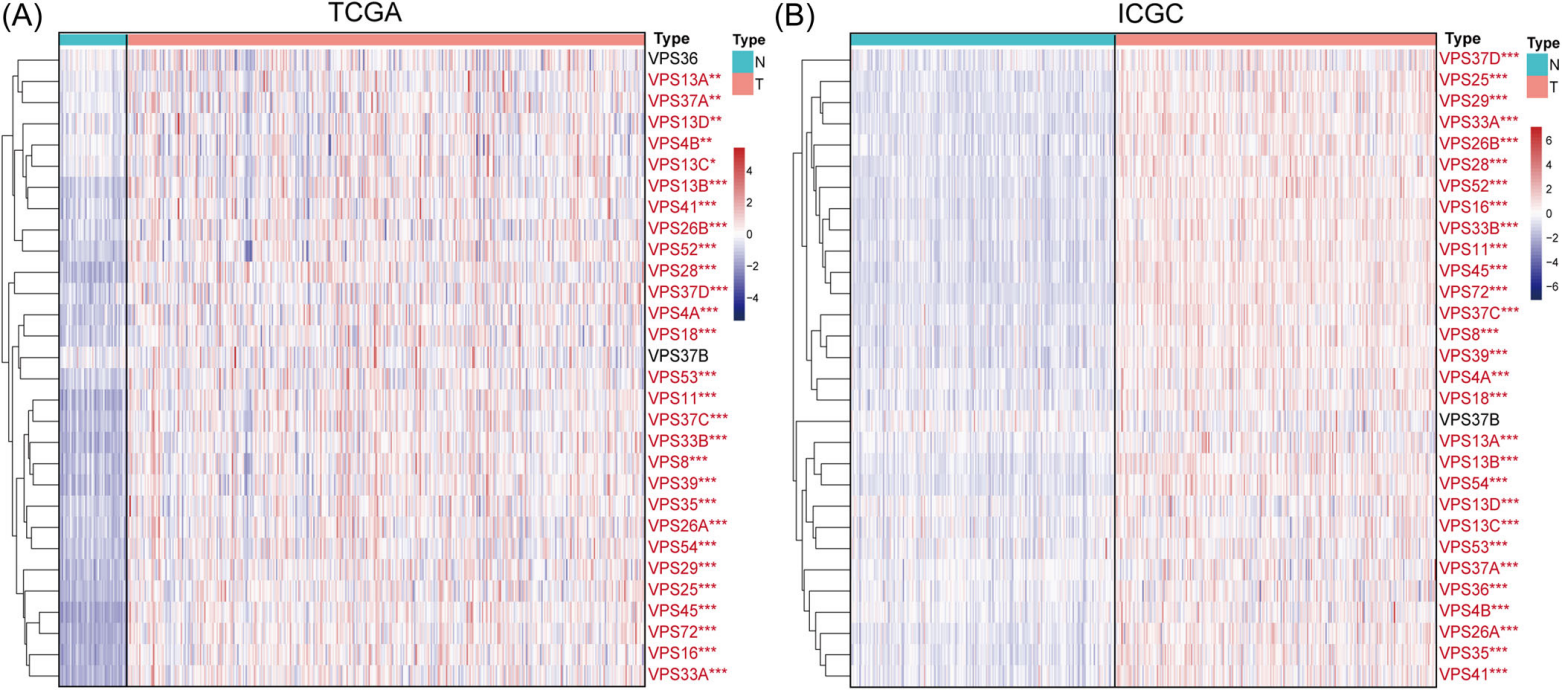
Transcriptional levels of VPSs family between HCC and normal tissues in TCGA (A) and ICGC (B). *p < .05, **p < .01, and ***p < .001. HCC, hepatocellular carcinoma; ICGC, The International Cancer Genome Consortium; VPS, vacuolar protein sorting; TCGA, The Cancer Genome Atlas.

Combining the analysis results of The Cancer Genome Atlas (TCGA) and International Cancer Genome Consortium (ICGC) databases revealed that VPS4A/4B/8/11/13A/13B/13C/13D/16/18/25/26A/26B/28/29/33A/33B/35/37A/37C/37D/39/41/45/52/53/54/72 was significantly overexpressed in HCC tissues more than in normal liver tissues (Figure 1A,B).

### 2.2 VPSs family gene mutation and correlation analysis

**Figure 2:**
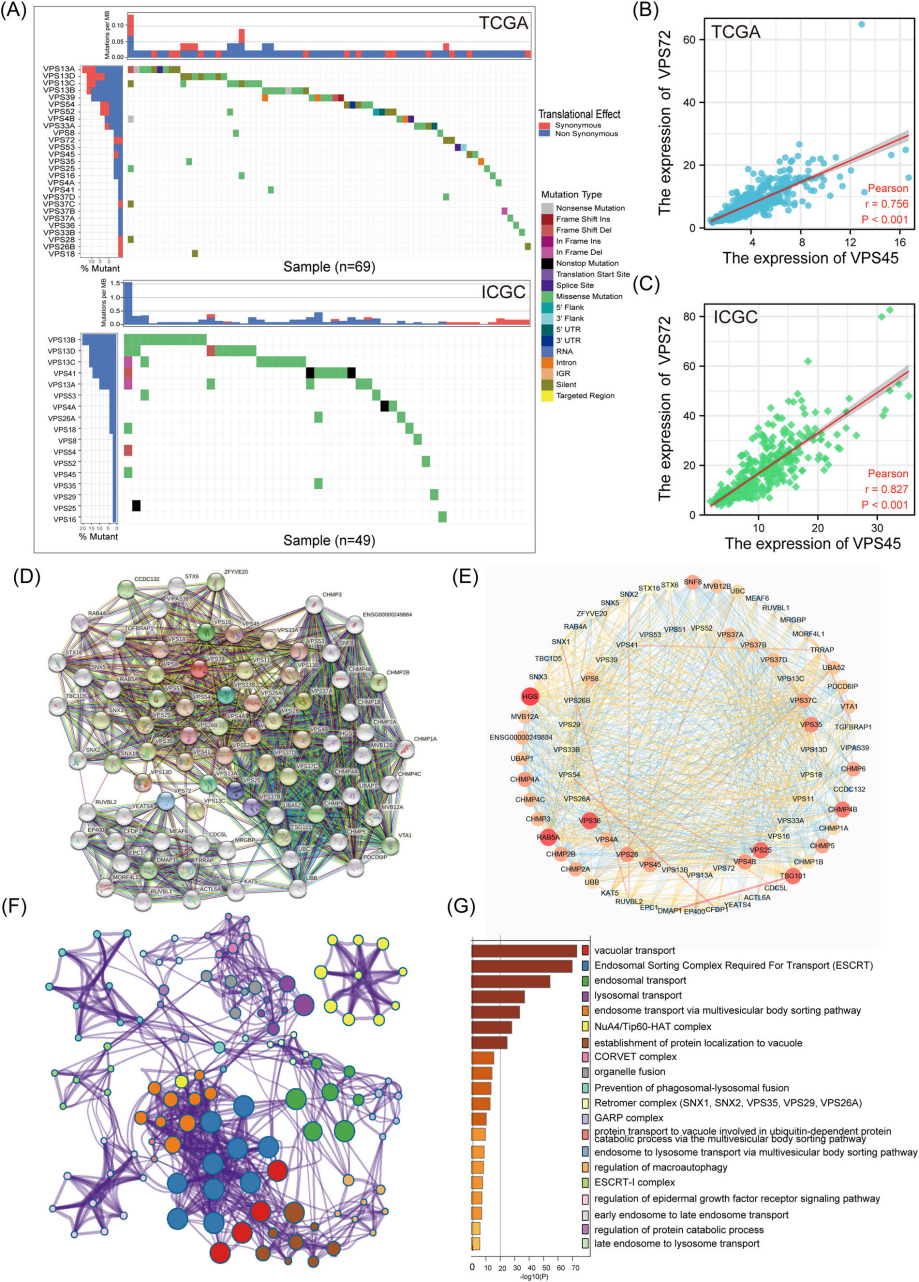
Analysis of correlation among VPSs, gene mutation, and functional enrichment analysis of VPSs. (A) The mutation rates of VPS13A, VPS13B, VPS13C, and VPS13D are all higher than 10%; missense mutation is the most common mutation type; among the VPS family genes, VPS45 and VPS72 have the highest correlation in TCGA (B) and ICGC (C) databases. (D) The neighbor genes significantly related to VPSs were found through the STRING database. (E) Using Cytoscape 3.7.1 to display PPI results through intergene connectivity, VPS25 and VPS72 played a central role in this network. (F, G) the analysis of annotation, visualization, and comprehensive discovery in the Metascape database. HCC, hepatocellular carcinoma; ICGC, The International Cancer Genome Consortium; PPI, protein–protein interaction; TCGA, The Cancer Genome Atlas; VPS, vacuolar protein sorting.

Thirty VPS mutations and their relationships were studied using somatic mutation and expression data from VPSs. First, based on the results of the VPSs genetic mutation, the VPS13 gene mutation rate was higher in HCC patients, primarily due to missense mutations (Figure 2A). Then, based on the results of the expression correlation (Supporting Information: Figure S1), the most significant correlation between VPS45 and VPS72 was 0.76 (Figure 2B) in TCGA and 0.83 in the ICGC database (Figure 2C). Protein–protein interaction (PPI) analysis revealed that 49 neighbor genes had a significant relation to VPSs in the STRING database (Figure 2D). Cytoscape 3.7.1 was utilized for visualizing PPI network results via the intergene connectivity, revealing that VPS25 and VPS36 exhibited a central part in this network, and there was a close relation between VPSs and CHMP families (Figure 2E). Through the Metascape database, “vacuolar transport” and “endosomal sorting complex required for transport (ESCRT)” are the main biological functions of these genes (Figure 2F,G).

### 2.3 Prognostic value and independent prognostic value of VPSs in HCC patients

**Figure 3:**
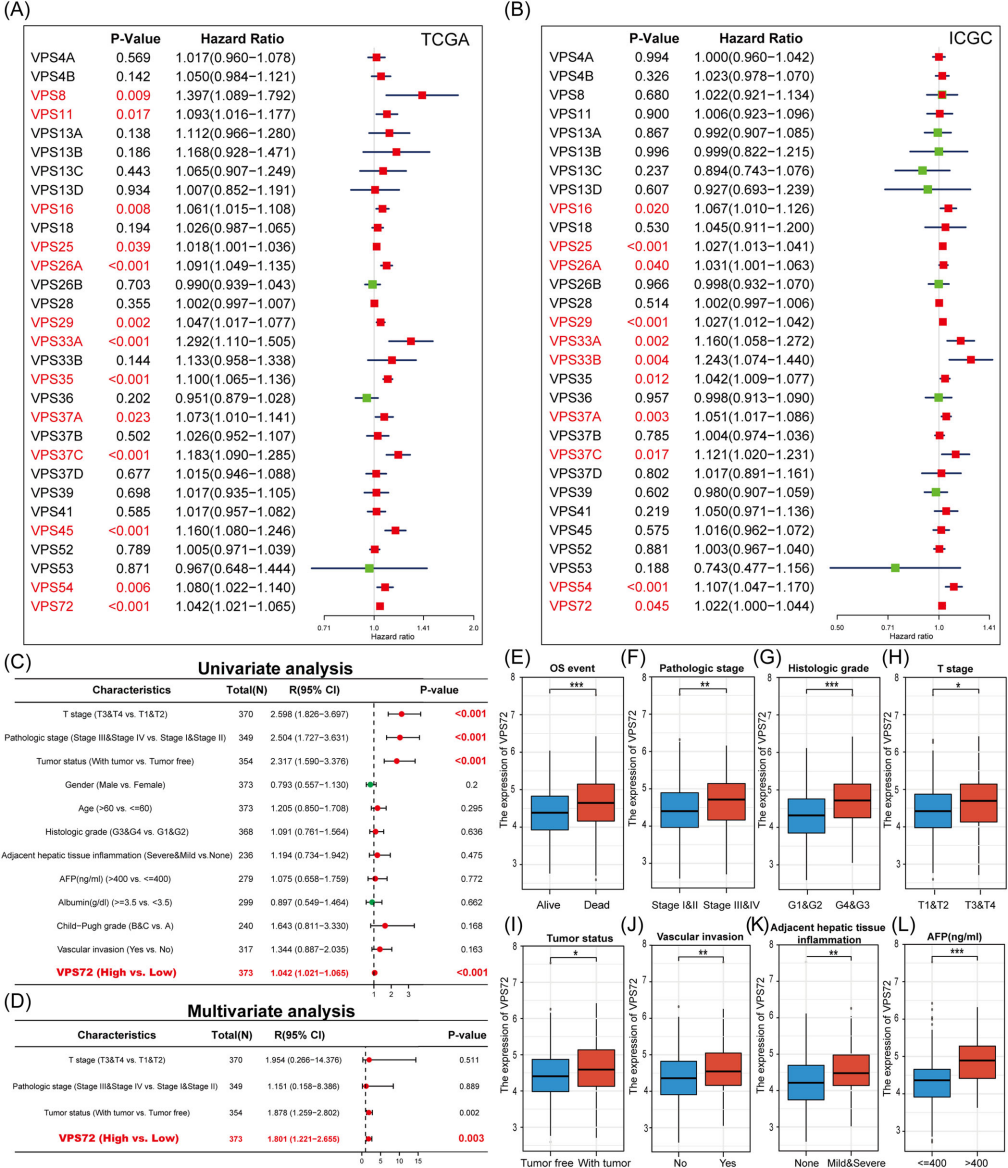
Prognostic value of different VPSs mRNA expression in hepatocellular carcinoma. (A, B) The mRNA expression level of VPSs was correlated with the OS in patients with HCC. Univariate and multivariate Cox regression analysis of the relationship between VPS72 and clinicopathological factors and OS of patients, The higher expression of VPS72 in patients with death. (E) and higher levels of the pathological stage (F), histologic grade (G), T stage (H), and AFP (L). Significant elevated VPS72 in HCC tissues of patients with postoperative recurrence (I) and vascular infiltration (J), and adjacent hepatic tissue inflammation (K). AFP, alpha‐fetoprotein; HCC, hepatocellular carcinoma; OS, overall survival; VPS, vacuolar protein sorting.

The relation between VPSs expression and patient OS was further analyzed in TCGA and ICGC databases, revealing that based on Univariate analysis, the higher VPS16/25/26A/29/33A/33B/37A/37C/54/72 mRNA expression levels were correlated to shorter OS in HCC (Figure 3A,B and Supporting Information: Figure S2), AS well as T stage, pathological stage, and tumor status, were correlated to shorter OS in HCC (Figure 3C). Moreover, based on further multivariate analysis, the higher VPS26A/33A/37C/72 mRNA expression was correlated to shorter OS in HCC patients (Supporting Information: Table S1), so VPS26A/33A/37C/72 have the potential to be independent risk factors for the purpose of predicting the prognosis of HCC patients, with lowest *p* value of 0.003 of VPS72 in multivariate Cox analysis (Figure 3D).

### 2.4 The association between VPS72 expression and clinicopathological factors in patients with HCC

VPS72 was discovered to have the ability to be an independent prognostic factor for predicting the prognosis of HCC patients through the studies described above. The association between VPS72 expression and clinicopathological factors in HCC patients was investigated, revealing that higher VPS72 expression in patients with death (Figure 3E) and higher levels of pathological stage (Figure 3F), histologic grade (Figure 3G), T stage (Figure 3H), and alpha‐fetoprotein (AFP) (Figure 3L). Significant elevated VPS72 in HCC tissues of patients with postoperative recurrence (Figure 3I) and vascular infiltration (Figure 3J), and adjacent hepatic tissue inflammation (Figure 3K).

**Figure 4:**
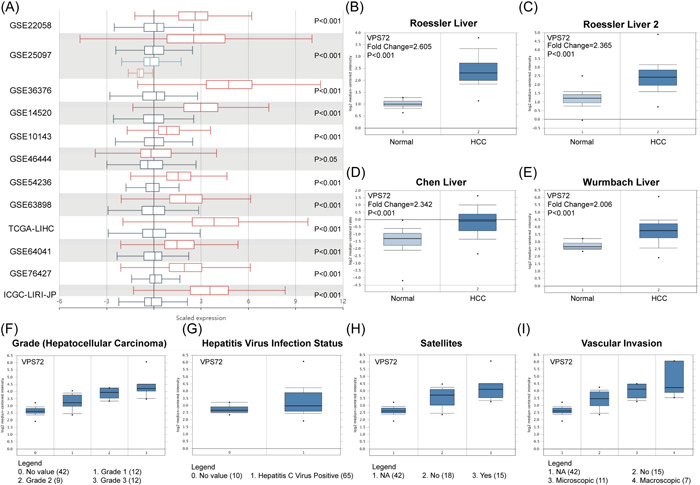
Relationship between VPS72 mRNA level and clinicopathological factors in other databases. (A) VPS72 mRNA level (HCCDB) in different HCC data sets. VPS72 was highly expressed in cancer tissues of HCC patients with multiple data sets in the ONCOMINE database. The fold change was 2.605 (B) in Roessler liver, 2.365 (C) in Roessler liver 2, and 2.342 (D) in Chen liver. The fold change in Wurmbach liver is 2.605 (E); in the Wambach data set, we found that the higher the expression of VPS72 in higher tumor grades (F), HCV positive (G), patients with satellites (H), and vascular invasion (I). HCC, hepatocellular carcinoma.

By analyzing multiple GEO chip data, the overexpressed VPS72 in HCC tissues (Figure 4A) was examined, revealing that VPS72 was overexpressed in cancer tissues of HCC patients with multiple data sets in the ONCOMINE database. The fold change was 2.605 (Figure 4B) in Roessler Liver, 2.365 (Figure 4C) in Roessler Liver 2, and 2.342 (Figure 4D) in Chen liver. The fold change in Wurmbach liver is 2.605 (Figure 4E); based on the Wambach data set, the higher VPS72 expression was found in higher tumor grades (Figure 4F), hepatitis C virus (HCV) positive (Figure 4G), patients with satellites (Figure 4H), and vascular invasion (Figure 4I).

### 2.5 Identification and analysis of VPS72‐related differentially expressed genes (DEGs)

**Figure 5:**
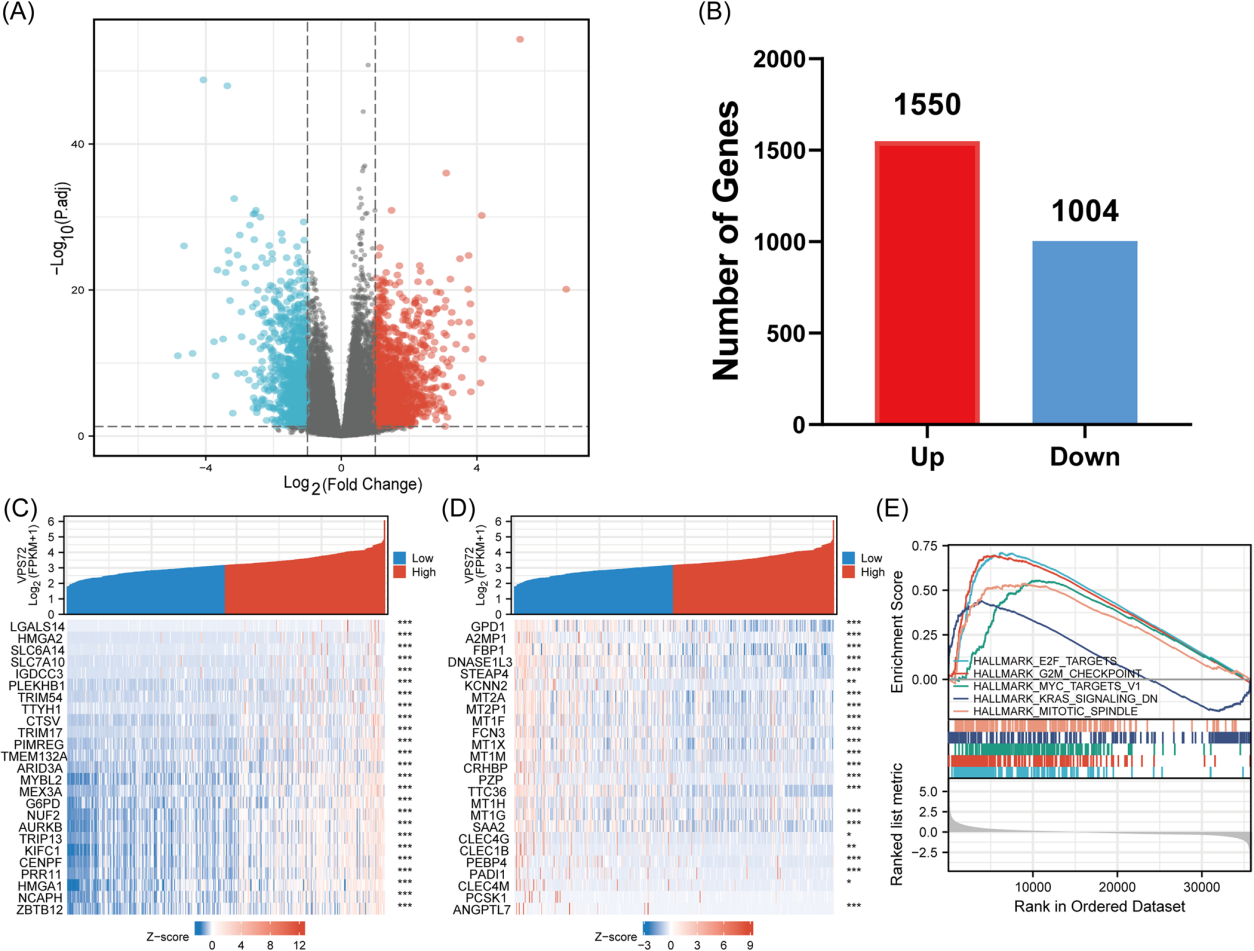
Volcano map and heat map of VPS72 high and VPS72 low expression in HCC samples. (A) the volcano map is described as 2554 DEGs (| log2fold change| > 1, adjusted *p* < .05). (B) the histogram of the number of upregulated or downregulated genes. (C, D) in HCC samples with high expression of VPS72 and low expression of VPS72, a heat map showed that 25 significantly upregulated and downregulated genes were correlated with the expression of VPS72. *p < .05, **p < .01, ***p < .001. (E) GSEA enrichment results of VPS72‐related DEGs. DEG, differentially expressed genes; GSEA, Gene Set Enrichment Analysis; HCC, hepatocellular carcinoma; VPS, vacuolar protein sorting.

For exploring the abnormal changes in the downstream signal transduction pathway resulting from VPS72 overexpression, the DEG were distinguished in HCC tissues with VPS72 overexpression and low expression according to TCGA data. Out of 2554 DEGs, 1550 were upregulated and 1004 downregulated. By generating Volcan diagrams and bar charts for the purpose of visually showing the DEGs distribution (Figure 5A,B), the heat map depicted the first 25 DEGs, which had significant upregulation and downregulation between VPS72 high‐ and low‐expression groups (Figure 5C,D). Through Gene Set Enrichment Analysis (GSEA) enrichment analysis, we found that multiple signal pathways affecting tumor progression were affected by VPS72 (Figure 5E).

### 2.6 Correlation analysis between VPS72 expression and various immune infiltrates

**Figure 6:**
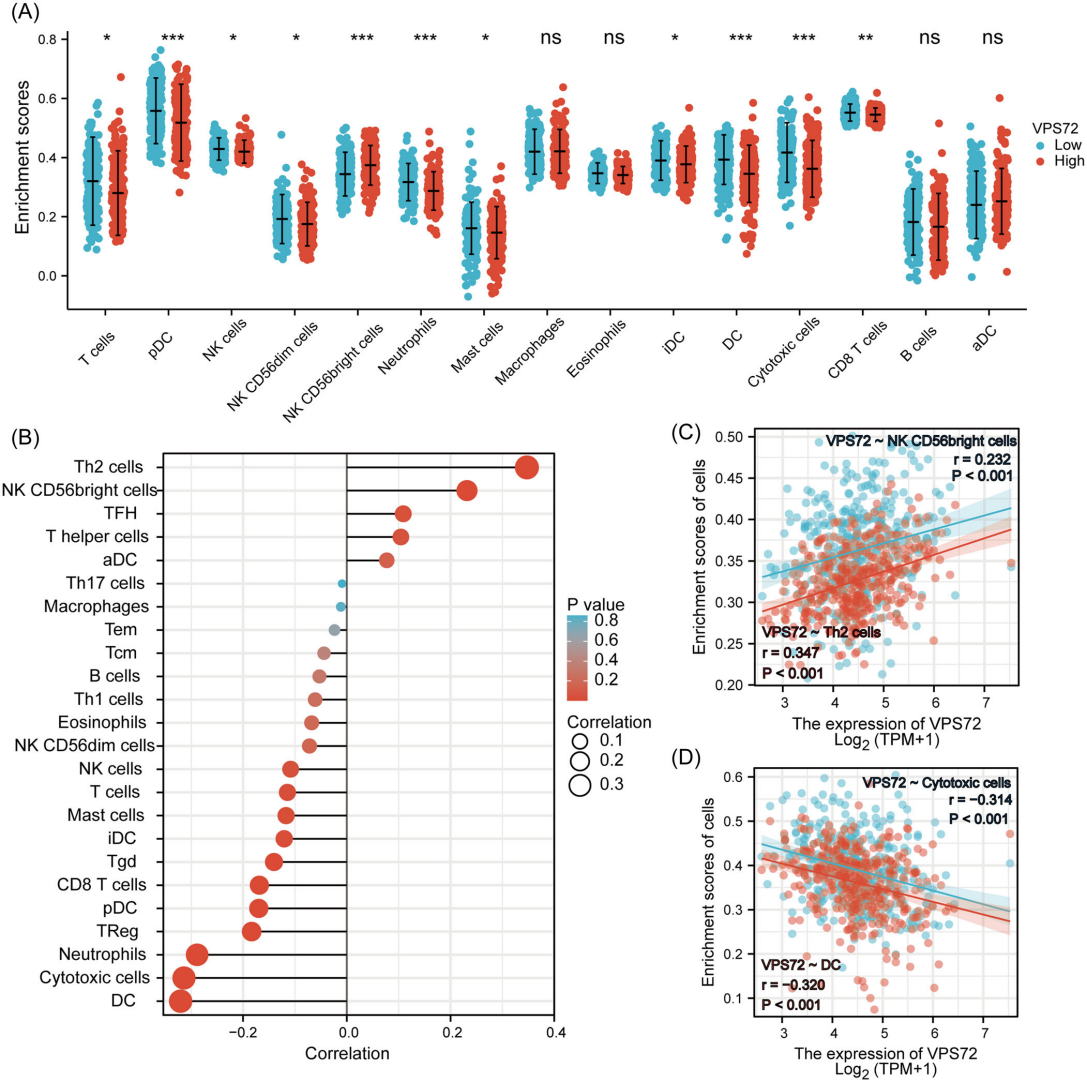
VPS72 expression and immune cell infiltration were detected by the TIMER database. (A) different proportions of immunocyte subtypes in HCC specimens of the VPS72 high expression group and VPS72 low expression group. (B, C) The correlation between the expression of VPS72 and 24 immune infiltrating cells. *p < .05, **p < .01, ***p < .001. HCC, hepatocellular carcinoma; VPS, vacuolar protein sorting.

The tumor microenvironment immune cells significantly affect the biological tumor behavior. VPS72 expression was discovered to have a correlation with the infiltration level of several immune cells in the HCC microenvironment by studying the infiltration of various immune cells (Figure 6A,B), which exhibited a positive correlation to Th2 cells, NKCD56 bright cell levels (Figure 6C), but negative correlation to innate immune cell levels such as dendritic cell and cytotoxic (Figure 6D).

## 3 DISCUSSION

HCC is caused by various factors, including metabolic syndrome, obesity, type 2 diabetes, and nonalcoholic fatty liver disease (NAFLD). It remains one of the deadliest malignant tumors worldwide.^17^ HCC occurrence and progression are linked to abnormal malignant cell events such as proliferation, migration, invasion, and autophagy.^18^, ^19^ Research has revealed that vesicle transport is closely related to cellular physiological processes like autophagy. VPS family proteins play an important role in vesicle transport^11^; however, VPS family proteins' role in diseases has not been thoroughly investigated. We have the lead in conducting this study to examine the expression, mutation, and prognostic value of different VPS family members in HCC.

Herein, 28 VPSs were significantly overexpressed in HCC patients. VPS16/25/26A/29/33A/33B/37A/37C/54/72 overexpression was positively correlated with short OS. Further univariate and multivariate analyses revealed that VPS26A/33A/37C/72 might serve as independent risk factors for the prognosis of HCC patients. According to other research, VPS33A is an important subunit of the class C VPS complex. Frameshift mutations in VPS33A may result in protein truncation and alter the interaction with the tumor suppressor UVRAG.^20^ VPS of melanoma cells with deletion of VPS33A or cappuccino protein (CNO) caused an increase in cis‐diaminedichloroplatinum II nuclear localization, DNA platinization damage, and apoptosis, thus improving the sensitivity of therapy.^21^ Chae et al. found that the decrease of VPS26A is related to synaptic defects, astrocyte overactivation, and cognitive impairment in diabetic mice.^22^ Eastman et al. proved that VPS37C is a functional component of mammalian ESCRT‐1.^23^ Furthermore, Kolmus et al. found that VPS37C expression is related to cellular stress response related to ESCRT‐1 instability, indicating that endocytosis is closely related to tumorigenesis and development,^24^ but how VPS33A, VPS26A, and VPS37C play a role in tumors needs further study.

At the end of mitosis, VPS72, also known as YL1, can mediate the nuclear recombination of H2A. VPS72 or H2A.Z deficiency caused malformed and dysfunctional nuclei.^25^, ^26^ Han et al. pointed out that VPS72 expression level may influence HCC biological process.^27^ Chris Kaiser et al. found that VPS72 is related to abnormal intracellular transport and abnormal vesicle morphology in *Saccharomyces cerevisiae*.^28^ In our study, through the analysis of TCGA and ICGC databases, VPS72 is overexpressed in HCC tissues, which has a correlation to a poor prognosis in HCC patients and may serve as an independent risk factor for HCC, and VPS72 is also highly expressed in HCC tissues in GEO database and multiple ONCOMINE data sets.

Moreover, VPS72 overexpression had a significant correlation to poor prognosis, higher Pathological stage, histologic grade, T stage, AFP level, postoperative recurrence, vascular infiltration, and the degree of adjacent hepatic tissue inflammation in HCC patients; moreover, VPS72 expression in ONCOMINE database was associated with higher tumor grades, patients with satellites, HCV positive, and vascular invasion. GSEA enrichment analysis demonstrated that VPS72 was related to E2F,^29^ G2M,^30^ MYC,^31^ and other signal pathways that affect tumor progression. Therefore, we believe VPS72 can affect HCC occurrence and progression and serve as a target for HCC diagnosis and treatment.

Recently, increasing evidence supports that the immune microenvironment affects HCC occurrence and development, which have adverse effects on the clinical prognosis and immunotherapy efficacy.^32^ A further major result of this study is the association between VPS72 mRNA level and immune cell infiltration degree in HCC. The levels of DC, cytotoxic cells, neutrophils, and especially Th2 cell had a significant correlation to VPS72 expression. Essential immunomodulatory cells in the body are Th cells; under normal circumstances, Th1/Th2 ratio is in a dynamic balance state.^33^ The increased Th2 cytokines secretion by patients having malignant tumors leads to the imbalance of Th1/Th2, which may be associated with the immune escape of many kinds of tumors,^34^ such as liver cancer.^35^ Therefore, VPS72 may influence the efficacy of immunotherapy in patients with liver cancer by affecting immune cell infiltration, but more research is required.

Our study has limitations, and more research is needed for the purpose of investigating the potential molecular mechanism of carcinogenesis of VPS family proteins. Nonetheless, we are the first to report the differential expression of the VPS protein family in HCC and its potential diagnostic and prognostic value. We observed that the overexpression of VPS protein in HCC was correlated to a poor prognosis. Further analysis displays that VPS26A/33A/37C/72 may serve as independent risk factors for determining patient prognosis. VPS72 overexpression has a positive correlation to various clinicopathological factors with poor prognosis and with the infiltration level of immune cells; hence, herein, the VPS family was found to have the ability to participate in HCC occurrence and development, especially VPS72, which may serve as a potential target for HCC treatment and prognostic biomarkers.

## 4 MATERIALS AND METHODS

### 4.1 Clinical samples and data collection

TCGA^36^ synapse data portal (https://cancergenome.nih.gov/), LIHC (liver hepatocellular carcinoma) was accessed for downloading RNAseq and mutation data and also extracting the clinical data of the patients (Table 1). The ICGC^37^ synapse data portal was also accessed to download RNAseq and mutation data for HCC samples.

**Table 1:** The basic characteristics of HCC patients.

Characteristics	HCC patient number
T stage	
T1	183
T2	95
T3	80
T4	13
N stage	
N0	254
N1	4
M stage	
M0	268
M1	4
Pathologic stage	
Stage I	173
Stage II	87
Stage III	85
Stage IV	5
Tumor status	
Tumor free	202
With tumor	153
Gender	
Female	121
Male	253
Age	
≤60	177
>60	196
Histologic grade	
G1	55
G2	178
G3	124
G4	12
Adjacent hepatic tissue inflammation	
None	118
Mild	101
Severe	18
AFP (ng/mL)	
≤400	215
>400	65
Albumin (g/dL)	
<3.5	69
≥3.5	231
Child‐Pugh grade	
A	219
B	21
C	1
Vascular invasion	
No	208
Yes	110

Abbreviations: AFP, alpha‐fetoprotein; HCC, hepatocellular carcinoma.

### 4.2 VPS family genes mutation and correlation analysis

The GenVisR^38^ package in R software was first used for analyzing VPS gene mutations in patients with HCC in TCGA and ICGC databases. The corrplot package was then used for examining the correlation between VPS genes in HCC patients.

### 4.3 Construction and functional enrichment analysis of VPSs protein‐related interaction network

STRING v11 ^39^(https://string-db.org/) was utilized to find 49 genes of VPS‐related genes. These networks can reveal multiple direct or indirect interactions associated with VPS genes. Cytoscape version 3.7.1^40^ was utilized for displaying the networks and hiding network disconnected nodes. The online analysis tool Metascape^41^ offers a comprehensive list of VPS‐related genes, VPS genes, and resources for real‐time analysis; pathway analyses were performed using Metascape.

### 4.4 Analysis of survival rate of VPSs

Most HCC patients have a poor prognosis, so OS was selected as the survival outcome. The survival package in R software was utilized for univariate Cox analysis, screening out the survival‐related VPSs (*p* < .05). Furthermore, multivariate Cox analysis was used for analyzing the VPSs significantly related to OS and its relationship with clinicopathological factors.

### 4.5 Other database validation

For validation, HCCDB was utilized for analyzing VPS72 expression in HCC. ONCOMINE database^42^ (www.oncomine.org) was then accessed to obtain VPS72 transcriptional expression between various cancer tissues and their corresponding normal control samples. Further analysis of the expression of VPS72 in different clinicopathological factors like grade, HCV positive, patients with satellites, and vascular invasion.

### 4.6 Functional enrichment analysis of VPS72 and its relationship with tumor immune microenvironment

Based on VPS72 expression levels, the gene expression data were categorized into high and low VPS72 groups. GSEA was conducted with the GSEA software. Spearman correlation analysis was used for evaluating the association between VPS72 expression and immune cell infiltration. Moreover, using the Wilcoxon rank sum test, the immune cell level of different VPS72 expression groups were analyzed.

### 4.7 Statistical analysis

R v4.1.2 (https://www.r-project.org/) and SPSS 26.0 (SPSS Inc.) were utilized for all statistical analyses. The Student's *t* test or one‐way analysis of variance (ANOVA) test was conducted for statistical analyses. Correlations of VPSs were evaluated with Spearman's correlation coefficient. To compare the H‐scores of HCC and normal tissues, paired *t* tests were used. *p < .05, **p < .01, and ***p < .001.

## AUTHOR CONTRIBUTIONS

**Jian Huang**: Data curation. **Jin Gan**: Formal analysis; visualization. **Jian Wang**: Methodology; writing—review and editing. **Min Zheng**: Writing—review and editing. **Han Xiao**: Conceptualization; data curation; formal analysis.

## CONFLICT OF INTEREST STATEMENT

The authors declare no competing interests.

## Supporting information

Supporting information.

**Figure S1**.

**Figure S2**.

## Data Availability

We obtained transcriptome data set and clinicopathological data set from TCGA (https://www.cancer.gov/tcga) and ICGC (https://icgc.org/) databases.

## References

[1] Sung H , Ferlay J , Siegel RL , et al. Global cancer statistics 2020: GLOBOCAN estimates of incidence and mortality worldwide for 36 cancers in 185 countries. CA Cancer J Clin. 2021;71(3):209‐249. 10.3322/caac.21660 33538338

[2] Anwanwan D , Singh SK , Singh S , Saikam V , Singh R . Challenges in liver cancer and possible treatment approaches. Biochim Biophys Acta Rev Cancer. 2020;1873(1):188314. 10.1016/j.bbcan.2019.188314 31682895 PMC6981221

[3] Sangro B , Sarobe P , Hervás‐Stubbs S , Melero I . Advances in immunotherapy for hepatocellular carcinoma. Nat Rev Gastroenterol Hepatol. 2021;18(8):525‐543. 10.1038/s41575-021-00438-0 33850328 PMC8042636

[4] Kelley RK , Greten TF . Hepatocellular carcinoma—origins and outcomes. N Engl J Med. 2021;385(3):280‐282. 10.1056/NEJMcibr2106594 34260842

[5] Llovet JM , Kelley RK , Villanueva A , et al. Hepatocellular carcinoma. Nat Rev Dis Primers. 2021;7(1):6. 10.1038/s41572-020-00240-3 33479224 PMC13537433

[6] Mellman I , Warren G . The road taken. Cell. 2000;100(1):99‐112. 10.1016/s0092-8674(00)81687-6 10647935

[7] Blott EJ , Griffiths GM . Secretory lysosomes. Nat Rev Mol Cell Biol. 2002;3(2):122‐131. 10.1038/nrm732 11836514

[8] Gagnon E , Duclos S , Rondeau C , et al. Endoplasmic reticulum‐mediated phagocytosis is a mechanism of entry into macrophages. Cell. 2002;110(1):119‐131. 10.1016/s0092-8674(02)00797-3 12151002

[9] Luzio JP , Poupon V , Lindsay MR , Mullock BM , Piper RC , Pryor PR . Membrane dynamics and the biogenesis of lysosomes. Mol Membr Biol. 2003;20(2):141‐154. 10.1080/0968768031000089546 12851071

[10] Pryor PR , Luzio JP . Delivery of endocytosed membrane proteins to the lysosome. Biochim Biophys Acta. 2009;1793(4):615‐624. 10.1016/j.bbamcr.2008.12.022 19167432

[11] Richardson SCW , Winistorfer SC , Poupon V , Luzio JP , Piper RC . Mammalian late vacuole protein sorting orthologues participate in early endosomal fusion and interact with the cytoskeleton. Mol Biol Cell. 2004;15(3):1197‐1210. 10.1091/mbc.e03-06-0358 14668490 PMC363107

[12] Segala G , Bennesch MA , Ghahhari NM , et al. Vps11 and Vps18 of Vps‐C membrane traffic complexes are E3 ubiquitin ligases and fine‐tune signalling. Nat Commun. 2019;10(1):1833. 10.1038/s41467-019-09800-y 31015428 PMC6478910

[13] Chen S , Mari M , Parashar S , et al. Vps13 is required for the packaging of the ER into autophagosomes during ER‐phagy. Proc Natl Acad Sci USA. 2020;117(31):18530‐18539. 10.1073/pnas.2008923117 32690699 PMC7414049

[14] Sanderson LE , Lanko K , Alsagob M , et al. Bi‐allelic variants in HOPS complex subunit VPS41 cause cerebellar ataxia and abnormal membrane trafficking. Brain. 2021;144(3):769‐780. 10.1093/brain/awaa459 33764426 PMC8041041

[15] Zhang J , Lin Y , Hu X , Wu Z , Guo W . VPS52 induces apoptosis via cathepsin D in gastric cancer. J Mol Med. 2017;95(10):1107‐1116. 10.1007/s00109-017-1572-y 28791438

[16] Wang C , Cheng Y , Zhang X , et al. Vacuolar protein sorting 33B is a tumor suppressor in hepatocarcinogenesis. Hepatology. 2018;68(6):2239‐2253. 10.1002/hep.30077 29729199

[17] Kulik L , El‐Serag HB . Epidemiology and management of hepatocellular carcinoma. Gastroenterology. 2019;156(2):477‐491.e1. 10.1053/j.gastro.2018.08.065 30367835 PMC6340716

[18] Jiang Y , Han Q , Zhao H , Zhang J . The mechanisms of HBV‐Induced hepatocellular carcinoma. J Hepatocell Carcinoma. 2021;8:435‐450. 10.2147/jhc.S307962 34046368 PMC8147889

[19] Chidambaranathan‐Reghupaty S , Fisher PB , Sarkar D . Hepatocellular carcinoma (HCC): epidemiology, etiology and molecular classification. Adv Cancer Res. 2021;149:1‐61. 10.1016/bs.acr.2020.10.001 33579421 PMC8796122

[20] Liang C , Lee J , Inn KS , et al. Beclin1‐binding UVRAG targets the class C Vps complex to coordinate autophagosome maturation and endocytic trafficking. Nature Cell Biol. 2008;10(7):776‐787. 10.1038/ncb1740 18552835 PMC2878716

[21] Huang Z , Chinen M , Chang PJ , et al. Targeting protein‐trafficking pathways alters melanoma treatment sensitivity. Proc Natl Acad Sci USA. 2012;109(2):553‐558. 10.1073/pnas.1118366109 22203954 PMC3258646

[22] Chae CW , Choi GE , Jung YH , et al. High glucose‐mediated VPS26a down‐regulation dysregulates neuronal amyloid precursor protein processing and tau phosphorylation. Br J Pharmacol. 2022;179:3934‐3950 10.1111/bph.15836 35297035

[23] Eastman SW , Martin‐Serrano J , Chung W , Zang T , Bieniasz PD . Identification of human VPS37C, a component of endosomal sorting complex required for transport‐I important for viral budding. J Biol Chem. 2005;280(1):628‐636. 10.1074/jbc.M410384200 15509564

[24] Kolmus K , Erdenebat P , Szymańska E , et al. Concurrent depletion of Vps37 proteins evokes ESCRT‐I destabilization and profound cellular stress responses. J Cell Sci. 2021;134(1):1‐19. 10.1242/jcs.250951 33419951

[25] Latrick CM , Marek M , Ouararhni K , et al. Molecular basis and specificity of H2A.Z‐H2B recognition and deposition by the histone chaperone YL1. Nat Struct Mol Biol. 2016;23(4):309‐316. 10.1038/nsmb.3189 26974126

[26] Moreno‐Andrés D , Yokoyama H , Scheufen A , et al. VPS72/YL1‐mediated H2A.Z deposition is required for nuclear reassembly after mitosis. Cells. 2020;9(7):1702. 10.3390/cells9071702 32708675 PMC7408173

[27] Han SS , Feng ZQ , Liu R , Ye J , Cheng WW . Bao J B. bioinformatics analysis and RNA‐sequencing of SCAMP3 expression and correlated gene regulation in hepatocellular carcinoma. Onco Targets Ther. 2020;13:1047‐1057. 10.2147/ott.S221785 32099407 PMC7007781

[28] Bonangelino CJ , Chavez EM , Bonifacino JS . Genomic screen for vacuolar protein sorting genes in *Saccharomyces cerevisiae* . Mol Biol Cell. 2002;13(7):2486‐2501. 10.1091/mbc.02-01-0005 12134085 PMC117329

[29] Kent LN , Leone G . The broken cycle: E2F dysfunction in cancer. Nat Rev Cancer. 2019;19(6):326‐338. 10.1038/s41568-019-0143-7 31053804

[30] Asghar U , Witkiewicz AK , Turner NC , Knudsen ES . The history and future of targeting cyclin‐dependent kinases in cancer therapy. Nat Rev Drug Discovery. 2015;14(2):130‐146. 10.1038/nrd4504 25633797 PMC4480421

[31] Duffy MJ , O'Grady S , Tang M , Crown J . MYC as a target for cancer treatment. Cancer Treat Rev. 2021;94:102154. 10.1016/j.ctrv.2021.102154 33524794

[32] Koon H , Atkins M . Autoimmunity and immunotherapy for cancer. N Engl J Med. 2006;354(7):758‐760. 10.1056/NEJMe058307 16481646

[33] Ding ZC , Blazar BR , Mellor AL , Munn DH , Zhou G . Chemotherapy rescues tumor‐driven aberrant CD4+ T‐cell differentiation and restores an activated polyfunctional helper phenotype. Blood. 2010;115(12):2397‐2406. 10.1182/blood-2009-11-253336 20118405 PMC2845898

[34] Ruterbusch M , Pruner KB , Shehata L , Pepper M . In vivo CD4(+) T cell differentiation and function: revisiting the Th1/Th2 paradigm. Annu Rev Immunol. 2020;38:705‐725. 10.1146/annurev-immunol-103019-085803 32340571

[35] Zhu Y , Yang J , Xu D , et al. Disruption of tumour‐associated macrophage trafficking by the osteopontin‐induced colony‐stimulating factor‐1 signalling sensitises hepatocellular carcinoma to anti‐PD‐L1 blockade. Gut. 2019;68(9):1653‐1666. 10.1136/gutjnl-2019-318419 30902885

[36] Liu J , Lichtenberg T , Hoadley KA , et al. An integrated TCGA Pan‐Cancer clinical data resource to drive high‐quality survival outcome analytics. Cell. 2018;173(2):400‐416.e11. 10.1016/j.cell.2018.02.052 29625055 PMC6066282

[37] Hudson TJ , Anderson W , Artez A , et al. International network of cancer genome projects. Nature. 2010;464(7291):993‐998. 10.1038/nature08987 20393554 PMC2902243

[38] Skidmore ZL , Campbell KM , Cotto KC , Griffith M , Griffith OL . Exploring the genomic landscape of cancer patient cohorts with GenVisR. Curr Protoc. 2021;1(9):e252. 10.1002/cpz1.252 34506690 PMC8439545

[39] Szklarczyk D , Gable AL , Lyon D , et al. STRING v11: protein‐protein association networks with increased coverage, supporting functional discovery in genome‐wide experimental datasets. Nucleic Acids Res. 2019;47(D1):D607‐D613. 10.1093/nar/gky1131 30476243 PMC6323986

[40] Shannon P , Markiel A , Ozier O , et al. Cytoscape: a software environment for integrated models of biomolecular interaction networks. Genome Res. 2003;13(11):2498‐2504. 10.1101/gr.1239303 14597658 PMC403769

[41] Zhou Y , Zhou B , Pache L , et al. Metascape provides a biologist‐oriented resource for the analysis of systems‐level datasets. Nat Commun. 2019;10(1):1523. 10.1038/s41467-019-09234-6 30944313 PMC6447622

[42] Rhodes DR , Yu J , Shanker K , et al. ONCOMINE: a cancer microarray database and integrated data‐mining platform. Neoplasia. 2004;6(1):1‐6. 10.1016/s1476-5586(04)80047-2 15068665 PMC1635162

