# Supplementary material for: VPS72, a member of VPS protein family, can be used as a new prognostic marker for hepatocellular carcinoma
Source: Immun Inflamm Dis. 2023 May 22;11(5):e856. doi: 10.1002/iid3.856 (PMC10201960; doi:10.1002/iid3.856)
Supplement: Supplementary file 1 — Supporting information. [file IID3-11-e856-s003.docx]

| **Characteristics** | **Total(N)** | **HR(95% CI) Univariate analysis** | **P value Univariate analysis** | **HR(95% CI) Multivariate analysis** | **P value Multivariate analysis** |
| --- | --- | --- | --- | --- | --- |
| T stage (T3&T4 vs. T1&T2) | 370 | 2.598 (1.826-3.697) | <0.001 | 1.642 (0.224-12.061) | 0.626 |
| Pathologic stage (Stage III&Stage IV vs. Stage I&Stage II) | 349 | 2.504 (1.727-3.631) | <0.001 | 1.311 (0.180-9.569) | 0.789 |
| Tumor status (With tumor vs. Tumor free) | 354 | 2.317 (1.590-3.376) | <0.001 | 1.826 (1.222-2.728) | 0.003 |
| Gender (Male vs. Female) | 373 | 0.793 (0.557-1.130) | 0.200 |  |  |
| Age (>60 vs. <=60) | 373 | 1.205 (0.850-1.708) | 0.295 |  |  |
| Histologic grade (G3&G4 vs. G1&G2) | 368 | 1.091 (0.761-1.564) | 0.636 |  |  |
| Adjacent hepatic tissue inflammation (Severe&Mild vs. None) | 236 | 1.194 (0.734-1.942) | 0.475 |  |  |
| AFP(ng/ml) (>400 vs. <=400) | 279 | 1.075 (0.658-1.759) | 0.772 |  |  |
| Albumin(g/dl) (>=3.5 vs. <3.5) | 299 | 0.897 (0.549-1.464) | 0.662 |  |  |
| Child-Pugh grade (B&C vs. A) | 240 | 1.643 (0.811-3.330) | 0.168 |  |  |
| Vascular invasion (Yes vs. No) | 317 | 1.344 (0.887-2.035) | 0.163 |  |  |
| VPS16 (High vs. Low) | 373 | 1.061 (1.015-1.108) | 0.008 | 1.340 (0.910-1.973) | 0.138 |
|  |  |  |  |  |  |
| **Characteristics** | **Total(N)** | **HR(95% CI) Univariate analysis** | **P value Univariate analysis** | **HR(95% CI) Multivariate analysis** | **P value Multivariate analysis** |
| T stage (T3&T4 vs. T1&T2) | 370 | 2.598 (1.826-3.697) | <0.001 | 1.721 (0.234-12.645) | 0.594 |
| Pathologic stage (Stage III&Stage IV vs. Stage I&Stage II) | 349 | 2.504 (1.727-3.631) | <0.001 | 1.248 (0.171-9.112) | 0.827 |
| Tumor status (With tumor vs. Tumor free) | 354 | 2.317 (1.590-3.376) | <0.001 | 1.789 (1.197-2.676) | 0.005 |
| Gender (Male vs. Female) | 373 | 0.793 (0.557-1.130) | 0.200 |  |  |
| Age (>60 vs. <=60) | 373 | 1.205 (0.850-1.708) | 0.295 |  |  |
| Histologic grade (G3&G4 vs. G1&G2) | 368 | 1.091 (0.761-1.564) | 0.636 |  |  |
| Adjacent hepatic tissue inflammation (Severe&Mild vs. None) | 236 | 1.194 (0.734-1.942) | 0.475 |  |  |
| AFP(ng/ml) (>400 vs. <=400) | 279 | 1.075 (0.658-1.759) | 0.772 |  |  |
| Albumin(g/dl) (>=3.5 vs. <3.5) | 299 | 0.897 (0.549-1.464) | 0.662 |  |  |
| Child-Pugh grade (B&C vs. A) | 240 | 1.643 (0.811-3.330) | 0.168 |  |  |
| Vascular invasion (Yes vs. No) | 317 | 1.344 (0.887-2.035) | 0.163 |  |  |
| VPS25 (High vs. Low) | 373 | 1.018(1.001-1.036) | 0.039 | 1.465 (0.993-2.161) | 0.054 |
|  |  |  |  |  |  |
| **Characteristics** | **Total(N)** | **HR(95% CI) Univariate analysis** | **P value Univariate analysis** | **HR(95% CI) Multivariate analysis** | **P value Multivariate analysis** |
| T stage (T3&T4 vs. T1&T2) | 370 | 2.598 (1.826-3.697) | <0.001 | 1.764 (0.240-12.959) | 0.577 |
| Pathologic stage (Stage III&Stage IV vs. Stage I&Stage II) | 349 | 2.504 (1.727-3.631) | <0.001 | 1.242 (0.170-9.048) | 0.831 |
| Tumor status (With tumor vs. Tumor free) | 354 | 2.317 (1.590-3.376) | <0.001 | 1.776 (1.186-2.659) | 0.005 |
| Gender (Male vs. Female) | 373 | 0.793 (0.557-1.130) | 0.200 |  |  |
| Age (>60 vs. <=60) | 373 | 1.205 (0.850-1.708) | 0.295 |  |  |
| Histologic grade (G3&G4 vs. G1&G2) | 368 | 1.091 (0.761-1.564) | 0.636 |  |  |
| Adjacent hepatic tissue inflammation (Severe&Mild vs. None) | 236 | 1.194 (0.734-1.942) | 0.475 |  |  |
| AFP(ng/ml) (>400 vs. <=400) | 279 | 1.075 (0.658-1.759) | 0.772 |  |  |
| Albumin(g/dl) (>=3.5 vs. <3.5) | 299 | 0.897 (0.549-1.464) | 0.662 |  |  |
| Child-Pugh grade (B&C vs. A) | 240 | 1.643 (0.811-3.330) | 0.168 |  |  |
| Vascular invasion (Yes vs. No) | 317 | 1.344 (0.887-2.035) | 0.163 |  |  |
| VPS26A (High vs. Low) | 373 | 1.091 (1.049-1.135) | <0.001 | 1.552 (1.052-2.290) | 0.027 |
|  |  |  |  |  |  |
| **Characteristics** | **Total(N)** | **HR(95% CI) Univariate analysis** | **P value Univariate analysis** | **HR(95% CI) Multivariate analysis** | **P value Multivariate analysis** |
| T stage (T3&T4 vs. T1&T2) | 370 | 2.598 (1.826-3.697) | <0.001 | 1.657 (0.225-12.184) | 0.620 |
| Pathologic stage (Stage III&Stage IV vs. Stage I&Stage II) | 349 | 2.504 (1.727-3.631) | <0.001 | 1.293 (0.177-9.451) | 0.800 |
| Tumor status (With tumor vs. Tumor free) | 354 | 2.317 (1.590-3.376) | <0.001 | 1.809 (1.208-2.710) | 0.004 |
| Gender (Male vs. Female) | 373 | 0.793 (0.557-1.130) | 0.200 |  |  |
| Age (>60 vs. <=60) | 373 | 1.205 (0.850-1.708) | 0.295 |  |  |
| Histologic grade (G3&G4 vs. G1&G2) | 368 | 1.091 (0.761-1.564) | 0.636 |  |  |
| Adjacent hepatic tissue inflammation (Severe&Mild vs. None) | 236 | 1.194 (0.734-1.942) | 0.475 |  |  |
| AFP(ng/ml) (>400 vs. <=400) | 279 | 1.075 (0.658-1.759) | 0.772 |  |  |
| Albumin(g/dl) (>=3.5 vs. <3.5) | 299 | 0.897 (0.549-1.464) | 0.662 |  |  |
| Child-Pugh grade (B&C vs. A) | 240 | 1.643 (0.811-3.330) | 0.168 |  |  |
| Vascular invasion (Yes vs. No) | 317 | 1.344 (0.887-2.035) | 0.163 |  |  |
| VPS29 (High vs. Low) | 373 | 1.047(1.047-1.077) | 0.002 | 1.348 (0.918-1.981) | 0.128 |
|  |  |  |  |  |  |
| **Characteristics** | **Total(N)** | **HR(95% CI) Univariate analysis** | **P value Univariate analysis** | **HR(95% CI) Multivariate analysis** | **P value Multivariate analysis** |
| T stage (T3&T4 vs. T1&T2) | 370 | 2.598 (1.826-3.697) | <0.001 | 1.739 (0.237-12.764) | 0.586 |
| Pathologic stage (Stage III&Stage IV vs. Stage I&Stage II) | 349 | 2.504 (1.727-3.631) | <0.001 | 1.213 (0.166-8.854) | 0.849 |
| Tumor status (With tumor vs. Tumor free) | 354 | 2.317 (1.590-3.376) | <0.001 | 1.830 (1.225-2.732) | 0.003 |
| Gender (Male vs. Female) | 373 | 0.793 (0.557-1.130) | 0.200 |  |  |
| Age (>60 vs. <=60) | 373 | 1.205 (0.850-1.708) | 0.295 |  |  |
| Histologic grade (G3&G4 vs. G1&G2) | 368 | 1.091 (0.761-1.564) | 0.636 |  |  |
| Adjacent hepatic tissue inflammation (Severe&Mild vs. None) | 236 | 1.194 (0.734-1.942) | 0.475 |  |  |
| AFP(ng/ml) (>400 vs. <=400) | 279 | 1.075 (0.658-1.759) | 0.772 |  |  |
| Albumin(g/dl) (>=3.5 vs. <3.5) | 299 | 0.897 (0.549-1.464) | 0.662 |  |  |
| Child-Pugh grade (B&C vs. A) | 240 | 1.643 (0.811-3.330) | 0.168 |  |  |
| Vascular invasion (Yes vs. No) | 317 | 1.344 (0.887-2.035) | 0.163 |  |  |
| VPS33A (High vs. Low) | 373 | 1.292(1.110-1.505) | <0.001 | 1.549 (1.051-2.284) | 0.027 |
|  |  |  |  |  |  |
| **Characteristics** | **Total(N)** | **HR(95% CI) Univariate analysis** | **P value Univariate analysis** | **HR(95% CI) Multivariate analysis** | **P value Multivariate analysis** |
| T stage (T3&T4 vs. T1&T2) | 370 | 2.598 (1.826-3.697) | <0.001 | 1.675 (0.228-12.296) | 0.612 |
| Pathologic stage (Stage III&Stage IV vs. Stage I&Stage II) | 349 | 2.504 (1.727-3.631) | <0.001 | 1.255 (0.172-9.174) | 0.823 |
| Tumor status (With tumor vs. Tumor free) | 354 | 2.317 (1.590-3.376) | <0.001 | 1.791 (1.197-2.681) | 0.005 |
| Gender (Male vs. Female) | 373 | 0.793 (0.557-1.130) | 0.200 |  |  |
| Age (>60 vs. <=60) | 373 | 1.205 (0.850-1.708) | 0.295 |  |  |
| Histologic grade (G3&G4 vs. G1&G2) | 368 | 1.091 (0.761-1.564) | 0.636 |  |  |
| Adjacent hepatic tissue inflammation (Severe&Mild vs. None) | 236 | 1.194 (0.734-1.942) | 0.475 |  |  |
| AFP(ng/ml) (>400 vs. <=400) | 279 | 1.075 (0.658-1.759) | 0.772 |  |  |
| Albumin(g/dl) (>=3.5 vs. <3.5) | 299 | 0.897 (0.549-1.464) | 0.662 |  |  |
| Child-Pugh grade (B&C vs. A) | 240 | 1.643 (0.811-3.330) | 0.168 |  |  |
| Vascular invasion (Yes vs. No) | 317 | 1.344 (0.887-2.035) | 0.163 |  |  |
| VPS35 (High vs. Low) | 373 | 1.100 (1.065-1.136) | <0.001 | 1.420 (0.965-2.092) | 0.075 |
|  |  |  |  |  |  |
| **Characteristics** | **Total(N)** | **HR(95% CI) Univariate analysis** | **P value Univariate analysis** | **HR(95% CI) Multivariate analysis** | **P value Multivariate analysis** |
| T stage (T3&T4 vs. T1&T2) | 370 | 2.598 (1.826-3.697) | <0.001 | 1.441 (0.197-10.512) | 0.719 |
| Pathologic stage (Stage III&Stage IV vs. Stage I&Stage II) | 349 | 2.504 (1.727-3.631) | <0.001 | 1.514 (0.209-10.967) | 0.681 |
| Tumor status (With tumor vs. Tumor free) | 354 | 2.317 (1.590-3.376) | <0.001 | 1.848 (1.237-2.761) | 0.003 |
| Gender (Male vs. Female) | 373 | 0.793 (0.557-1.130) | 0.200 |  |  |
| Age (>60 vs. <=60) | 373 | 1.205 (0.850-1.708) | 0.295 |  |  |
| Histologic grade (G3&G4 vs. G1&G2) | 368 | 1.091 (0.761-1.564) | 0.636 |  |  |
| Adjacent hepatic tissue inflammation (Severe&Mild vs. None) | 236 | 1.194 (0.734-1.942) | 0.475 |  |  |
| AFP(ng/ml) (>400 vs. <=400) | 279 | 1.075 (0.658-1.759) | 0.772 |  |  |
| Albumin(g/dl) (>=3.5 vs. <3.5) | 299 | 0.897 (0.549-1.464) | 0.662 |  |  |
| Child-Pugh grade (B&C vs. A) | 240 | 1.643 (0.811-3.330) | 0.168 |  |  |
| Vascular invasion (Yes vs. No) | 317 | 1.344 (0.887-2.035) | 0.163 |  |  |
| VPS37A (High vs. Low) | 373 | 1.073 (1.101-1.141) | 0.023 | 1.124 (1.065-1.171) | 0.143 |
|  |  |  |  |  |  |
| **Characteristics** | **Total(N)** | **HR(95% CI) Univariate analysis** | **P value Univariate analysis** | **HR(95% CI) Multivariate analysis** | **P value Multivariate analysis** |
| T stage (T3&T4 vs. T1&T2) | 370 | 2.598 (1.826-3.697) | <0.001 | 1.782 (0.243-13.080) | 0.570 |
| Pathologic stage (Stage III&Stage IV vs. Stage I&Stage II) | 349 | 2.504 (1.727-3.631) | <0.001 | 1.196 (0.164-8.727) | 0.860 |
| Tumor status (With tumor vs. Tumor free) | 354 | 2.317 (1.590-3.376) | <0.001 | 1.773 (1.185-2.653) | 0.005 |
| Gender (Male vs. Female) | 373 | 0.793 (0.557-1.130) | 0.200 |  |  |
| Age (>60 vs. <=60) | 373 | 1.205 (0.850-1.708) | 0.295 |  |  |
| Histologic grade (G3&G4 vs. G1&G2) | 368 | 1.091 (0.761-1.564) | 0.636 |  |  |
| Adjacent hepatic tissue inflammation (Severe&Mild vs. None) | 236 | 1.194 (0.734-1.942) | 0.475 |  |  |
| AFP(ng/ml) (>400 vs. <=400) | 279 | 1.075 (0.658-1.759) | 0.772 |  |  |
| Albumin(g/dl) (>=3.5 vs. <3.5) | 299 | 0.897 (0.549-1.464) | 0.662 |  |  |
| Child-Pugh grade (B&C vs. A) | 240 | 1.643 (0.811-3.330) | 0.168 |  |  |
| Vascular invasion (Yes vs. No) | 317 | 1.344 (0.887-2.035) | 0.163 |  |  |
| VPS37C (High vs. Low) | 373 | 1.734 (1.222-2.462) | 0.002 | 1.608 (1.091-2.371) | 0.016 |
|  |  |  |  |  |  |
| **Characteristics** | **Total(N)** | **HR(95% CI) Univariate analysis** | **P value Univariate analysis** | **HR(95% CI) Multivariate analysis** | **P value Multivariate analysis** |
| T stage (T3&T4 vs. T1&T2) | 370 | 2.598 (1.826-3.697) | <0.001 | 1.597 (0.217-11.749) | 0.646 |
| Pathologic stage (Stage III&Stage IV vs. Stage I&Stage II) | 349 | 2.504 (1.727-3.631) | <0.001 | 1.376 (0.189-10.034) | 0.753 |
| Tumor status (With tumor vs. Tumor free) | 354 | 2.317 (1.590-3.376) | <0.001 | 1.810 (1.209-2.710) | 0.004 |
| Gender (Male vs. Female) | 373 | 0.793 (0.557-1.130) | 0.200 |  |  |
| Age (>60 vs. <=60) | 373 | 1.205 (0.850-1.708) | 0.295 |  |  |
| Histologic grade (G3&G4 vs. G1&G2) | 368 | 1.091 (0.761-1.564) | 0.636 |  |  |
| Adjacent hepatic tissue inflammation (Severe&Mild vs. None) | 236 | 1.194 (0.734-1.942) | 0.475 |  |  |
| AFP(ng/ml) (>400 vs. <=400) | 279 | 1.075 (0.658-1.759) | 0.772 |  |  |
| Albumin(g/dl) (>=3.5 vs. <3.5) | 299 | 0.897 (0.549-1.464) | 0.662 |  |  |
| Child-Pugh grade (B&C vs. A) | 240 | 1.643 (0.811-3.330) | 0.168 |  |  |
| Vascular invasion (Yes vs. No) | 317 | 1.344 (0.887-2.035) | 0.163 |  |  |
| VPS54 (High vs. Low) | 373 | 1.080 (1.022-1.140) | 0.006 | 1.235 (0.834-1.827) | 0.292 |
|  |  |  |  |  |  |
| **Characteristics** | **Total(N)** | **HR(95% CI) Univariate analysis** | **P value Univariate analysis** | **HR(95% CI) Multivariate analysis** | **P value Multivariate analysis** |
| T stage (T3&T4 vs. T1&T2) | 370 | 2.598 (1.826-3.697) | <0.001 | 1.954 (0.266-14.376) | 0.511 |
| Pathologic stage (Stage III&Stage IV vs. Stage I&Stage II) | 349 | 2.504 (1.727-3.631) | <0.001 | 1.151 (0.158-8.386) | 0.889 |
| Tumor status (With tumor vs. Tumor free) | 354 | 2.317 (1.590-3.376) | <0.001 | 1.878 (1.259-2.802) | 0.002 |
| Gender (Male vs. Female) | 373 | 0.793 (0.557-1.130) | 0.200 |  |  |
| Age (>60 vs. <=60) | 373 | 1.205 (0.850-1.708) | 0.295 |  |  |
| Histologic grade (G3&G4 vs. G1&G2) | 368 | 1.091 (0.761-1.564) | 0.636 |  |  |
| Adjacent hepatic tissue inflammation (Severe&Mild vs. None) | 236 | 1.194 (0.734-1.942) | 0.475 |  |  |
| AFP(ng/ml) (>400 vs. <=400) | 279 | 1.075 (0.658-1.759) | 0.772 |  |  |
| Albumin(g/dl) (>=3.5 vs. <3.5) | 299 | 0.897 (0.549-1.464) | 0.662 |  |  |
| Child-Pugh grade (B&C vs. A) | 240 | 1.643 (0.811-3.330) | 0.168 |  |  |
| Vascular invasion (Yes vs. No) | 317 | 1.344 (0.887-2.035) | 0.163 |  |  |
| VPS72 (High vs. Low) | 373 | 1.042 (1.021-1.065) | <0.001 | 1.801 (1.221-2.655) | 0.003 |

**TableS1**. Univariate and multivariate analysis of 10 VPSs related to prognosis and clinicopathological factors.
