# Supplementary figures and images for: VPS72, a member of VPS protein family, can be used as a new prognostic marker for hepatocellular carcinoma
Source: Immun Inflamm Dis. 2023 May 22;11(5):e856. doi: 10.1002/iid3.856 (PMC10201960; doi:10.1002/iid3.856)

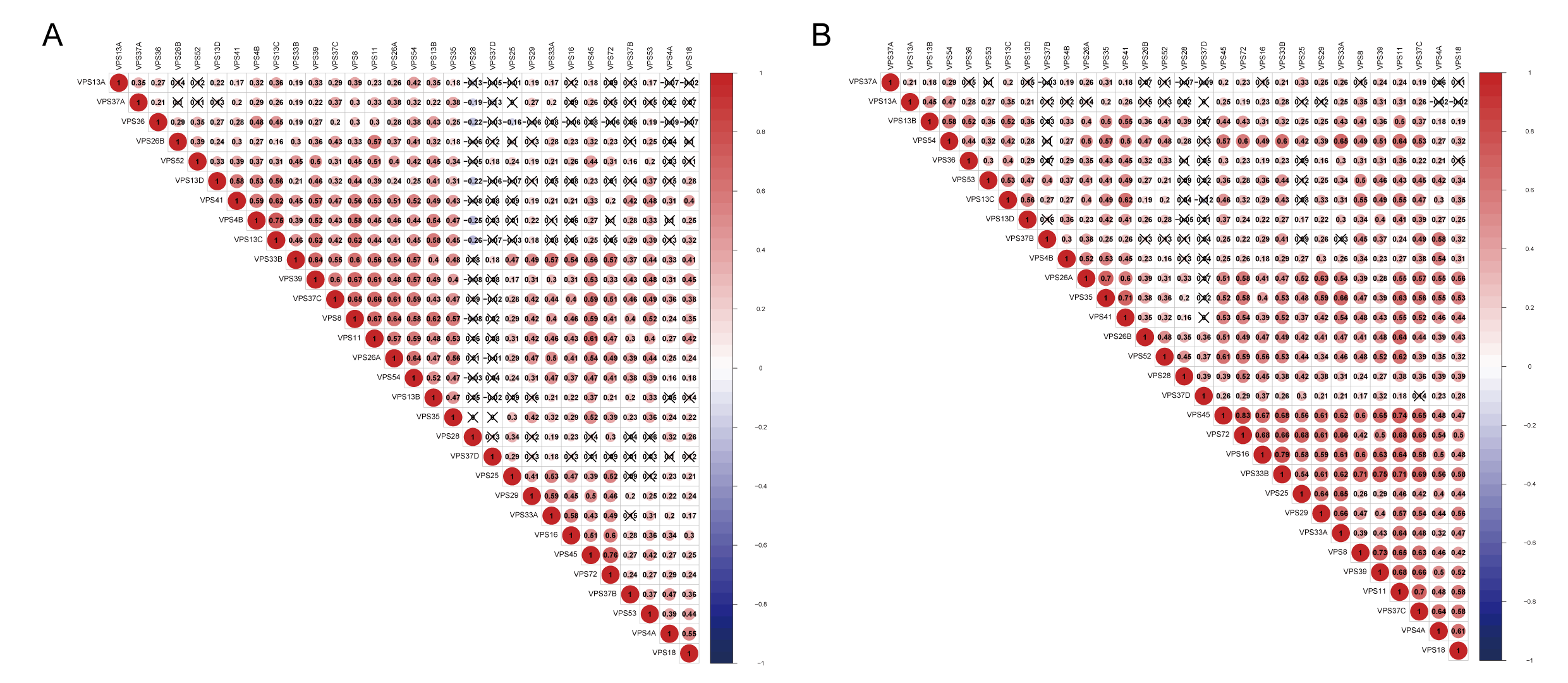

Supplement: Supplementary file 2 — Figure S1. [file IID3-11-e856-s001.tif]

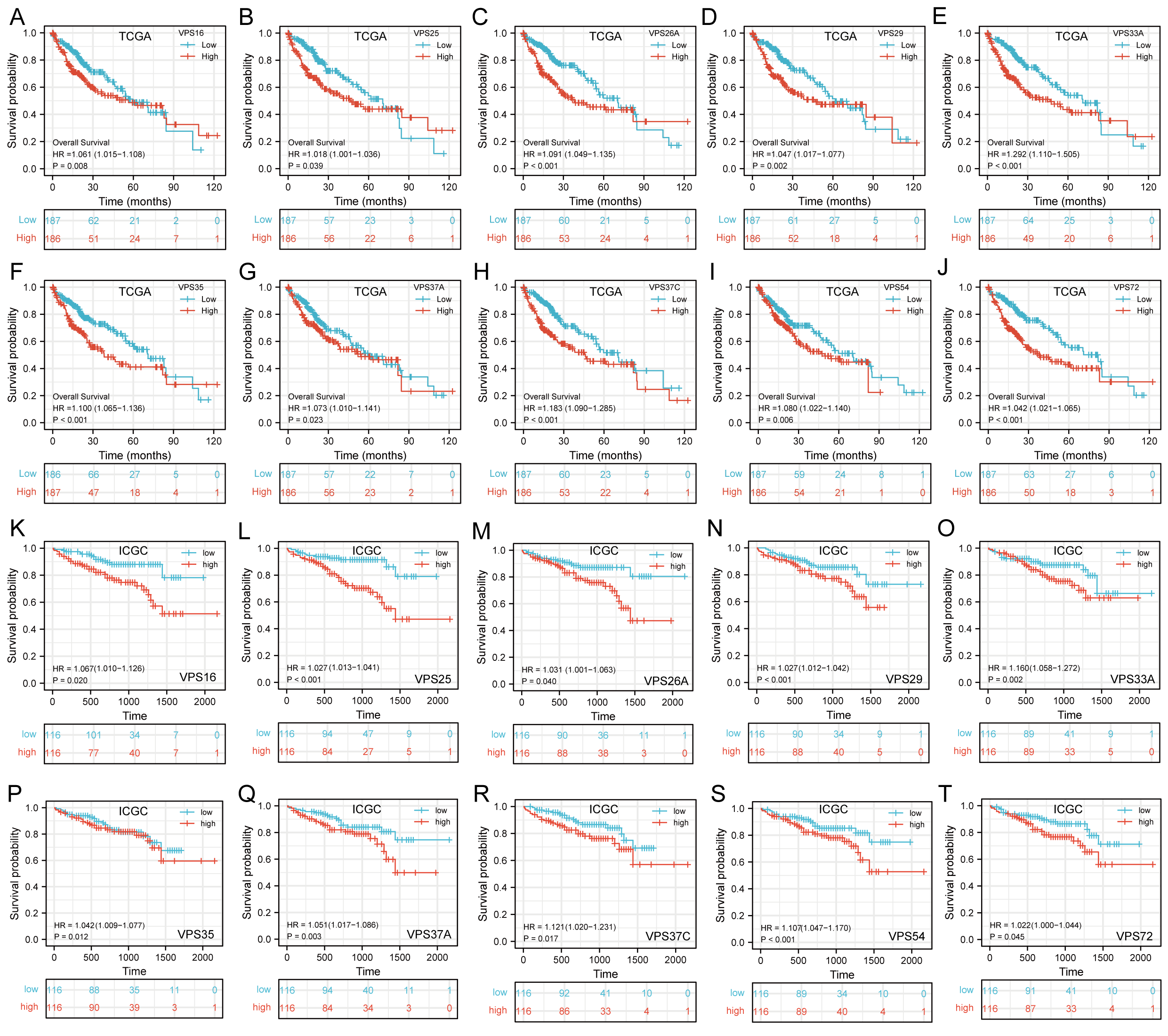

Supplement: Supplementary file 3 — Figure S2. [file IID3-11-e856-s002.tif]
